# Comparison of Weighted and Unweighted Population Data to Assess Inequities in Coronavirus Disease 2019 Deaths by Race/Ethnicity Reported by the US Centers for Disease Control and Prevention

**DOI:** 10.1001/jamanetworkopen.2020.16933

**Published:** 2020-07-28

**Authors:** Tori L. Cowger, Brigette A. Davis, Onisha S. Etkins, Keletso Makofane, Jourdyn A. Lawrence, Mary T. Bassett, Nancy Krieger

**Affiliations:** 1 FXB Center for Health and Human Rights, Harvard T.H. Chan School of Public Health, Boston, Massachusetts; 2 Department of Epidemiology, Harvard T.H. Chan School of Public Health, Boston, Massachusetts; 3 Population Health Sciences Department, Harvard Graduate School of Arts and Sciences, Cambridge, Massachusetts; 4 Department of Social and Behavioral Sciences, Harvard T.H. Chan School of Public Health, Boston, Massachusetts

## Abstract

This cross-sectional study compares the use of weighted and unweighted population data to assess inequities in coronavirus disease 2019 (COVID-19) deaths by race/ethnicity as reported by the US Centers for Disease Control and Prevention (CDC).

## Introduction

Surveillance and mortality data show large inequities in the impact of coronavirus disease 2019 (COVID-19) by race/ethnicity.^1^ Currently, the US Centers for Disease Control and Prevention (CDC) does not report mortality rates by race/ethnicity. Instead, the percentage distribution of COVID-19 deaths by race/ethnicity is presented alongside a weighted distribution of the population from the CDC’s National Center for Health Statistics,^2^ which weights each county’s population by its share of COVID-19 deaths, not population (Figure). We investigated whether the resulting magnitude of inequities using the weighted population underestimates those observed using the total population (unweighted).

**Figure.**
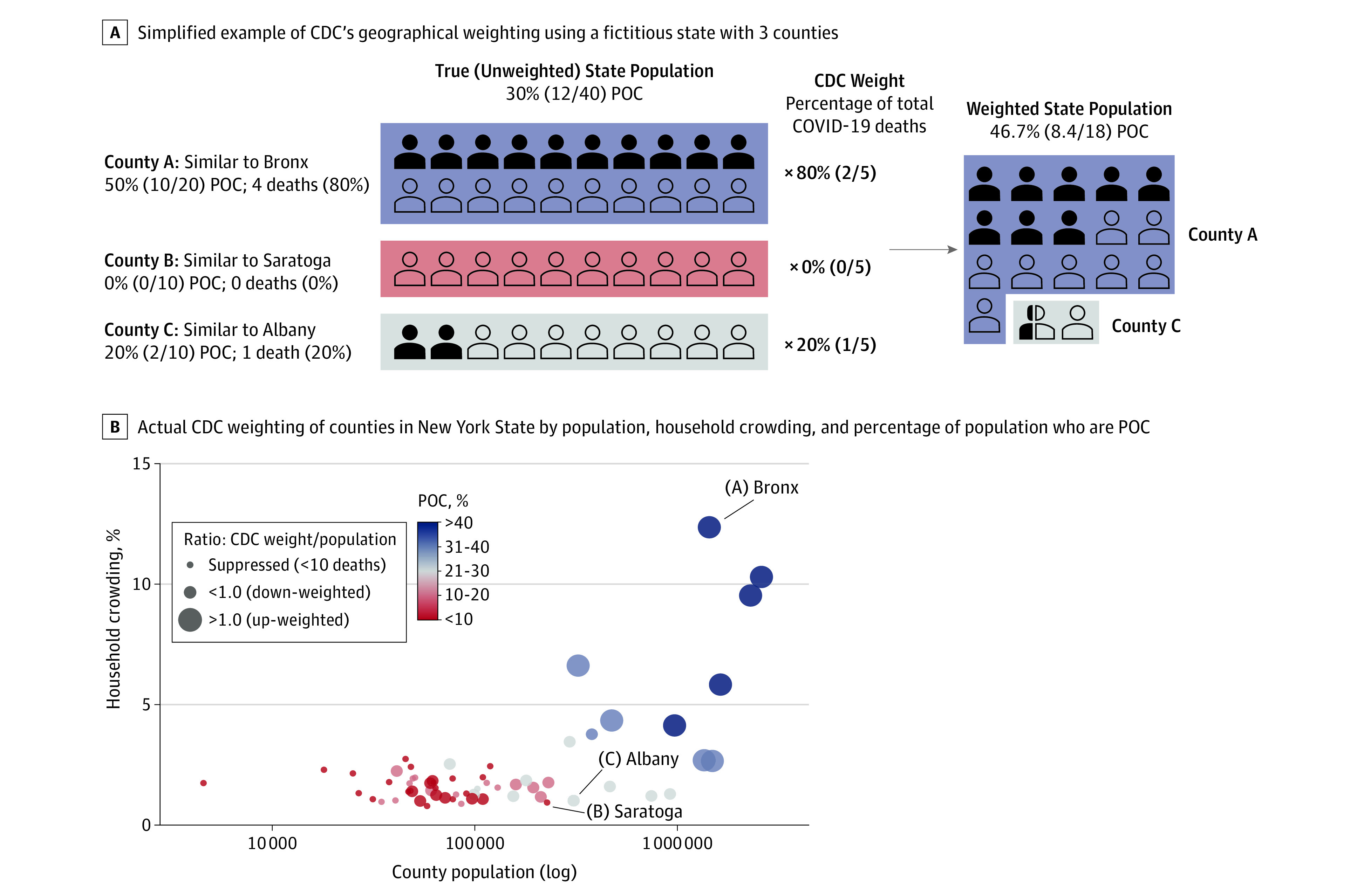
Examples of US Centers for Disease Control and Prevention (CDC) Geographical Population Weighting The figure shows examples of CDC geographical population weighting using a fictitious state with 3 counties (A) and actual CDC weighting of counties in New York State (B) by population, household crowding, and percentage of county population who are people of color (POC). The impact of the CDC’s method of geographical reweighting is demonstrated by juxtaposing the hypothetical example in panel A with actual county population data in panel B. By up-weighting counties such as county A (eg, Bronx), down-weighting counties such as county C (eg, Albany), and excluding counties such as county B (eg, Saratoga), the CDC inflates the proportion of residents of color in the weighted population, making their risk of death appear lower, while deflating the proportion of White residents, making their risk of death appear greater.

## Methods

This cross-sectional study used publicly available, aggregated data downloaded May 13, 2020.^2^ Because the data were deidentified, institutional review board approval and informed consent were not required, in accordance with 45 CFR §46. This study follows the relevant portions of the Strengthening the Reporting of Observational Studies in Epidemiology (STROBE) reporting guidelines.

We compared the distribution of COVID-19 deaths by race/ethnicity with 2 separate population distributions provided by the CDC: National Center for Health Statistics weighted population and US Census unweighted population. Data analysis was performed from May to June 2020 using R statistical software version 3.6.3 (R Project for Statistical Computing).

## Results

In total, 54 861 COVID-19 deaths were reported as of May 13, 2020. Applying the US Census population distribution, Black individuals were the most overrepresented among COVID-19 deaths, accounting for 9.9% greater than their share of the US Census population, whereas White individuals were underrepresented (−8.1%). In contrast, comparisons with the weighted data suggest that White individuals are most overrepresented among COVID-19 deaths (10.9%) (Table). Discrepancies were also noted when comparing deaths with the unweighted vs weighted populations among Latinx (−1.7% vs −10.2%) and Asian (0.1% vs −5.7%) individuals (Table).

**Table.** Percentage Distribution by Race/Ethnicity for COVID-19 Deaths, CDC-NCHS–Weighted Population, and US Census Population and Absolute and Relative Differences Using Data as of May 13, 2020

Race/ethnicity^a^	Distribution, %			Comparison with CDC-NCHS–weighted population		Comparison with US Census population (unweighted)	
	COVID-19 deaths^b^	CDC-NCHS–weighted population	US Census population	Difference, %^c^	Ratio^d^	Difference, %^e^	Ratio^f^
American Indian and Alaska Native^g^	0.4	0.2	0.7	0.2^h^	2.00^h^	−0.3	0.57
Asian American	5.8	11.5	5.7	−5.7	0.50	0.1^h^	1.02^h^
Black	22.4	18.2	12.5	4.2^h^	1.23^h^	9.9^h^	1.79^h^
Latinx	16.6	26.8	18.3	−10.2	0.62	−1.7	0.91
Other race^i^	2.5	1.9	2.4	0.6^h^	1.32^h^	0.1^h^	1.04^h^
White	52.3	41.4	60.4	10.9^h^	1.26^h^	−8.1	0.87

Abbreviations: CDC, Centers for Disease Control and Prevention; COVID-19, coronavirus disease 2019; NCHS, National Center for Health Statistics.

^a^ All racial/ethnic groups are shown directly as presented by the CDC in their weekly provisional death counts for COVID-19.

^b^ In total, 54 861 COVID-19 deaths were reported to the CDC as of May 13, 2020.

^c^ Percentage of COVID-19 deaths minus percentage CDC-NCHS–weighted population.

^d^ Percentage of COVID-19 deaths divided by percentage CDC-NCHS–weighted population.

^e^ Percentage of COVID-19 deaths minus percentage US Census population.

^f^ Percentage of COVID-19 deaths divided by percentage US Census population.

^g^ The American Indian and Alaska Native data should be viewed as likely inaccurate, given well-known issues with undercount of deaths and problems with US Census counts of these populations.

^h^ Indicates an excess in absolute or relative COVID-19 mortality compared with the population distribution (ie, overrepresentation among COVID-19 deaths).

^i^ Includes Native Hawaiian and other Pacific Islander, more than 1 race, race unknown, and Hispanic/Latinx origin unknown.

The CDC’s weighting approach inflates the proportion of residents of color in the weighted population, as shown in our hypothetical example in panel A of the Figure, where the state’s true population is 30% people of color, but the CDC’s weighted population is 46.7% people of color. For example, in New York, large urban counties with higher percentages of crowded households and residents of color are weighted more heavily compared with their share of the population than smaller, suburban, and rural counties, where residents are predominantly White, as shown in panel B of the Figure.

## Discussion

Use of the CDC’s weighted population distributions to evaluate racial/ethnic inequities in COVID-19 mortality underestimates the excess burden of COVID-19 among Black and Latinx individuals compared with analyses conducted using the total population (unweighted) in the US Census data. According to the CDC, weighting was conducted because “COVID-19 deaths are concentrated in certain geographic locations where the racial and ethnic population distribution differs from that of the United States overall.”^2^

The indirect standardization procedure implemented by the CDC is misleading and obviates a key mechanism by which structural racism operates to produce health inequities: social segregation.^3^ The CDC approach heavily weights large, urban counties because of their high proportion of COVID-19 deaths (eg, New York City) and excludes counties without any COVID-19 deaths (Figure). In effect, the CDC treats the geographical clustering of COVID-19 deaths as a nuisance parameter that must be controlled for to accurately compare the distribution of deaths across racial groups in the same geographical areas. However, the same mechanisms that pattern the geographical distribution of COVID-19 mortality also operate to produce racial/ethnic inequities in mortality.

From macrogeographical regions to microneighborhoods within cities, structural racism has determined the distribution of Black, Latinx, and Native American communities and is a key mechanism that produces and maintains inequities in infectious disease outcomes.^3,4,5^ Specifically, historical and contemporary policies and processes, including land theft, racial terrorism, redlining, and gentrification, determine the location, quality, and density of residence for people of color.^3,5^ Consequently, Black and Latinx individuals are clustered in the same high-density, urban locations hardest hit in the first months of the pandemic, with these areas weighted most heavily by the CDC’s procedure (Figure). By adjusting for the geographical distribution of racial groups, the CDC effectively compares inequities that would remain had all racial and ethnic groups lived in the same geographical areas. Controlling for this major pathway understates COVID-19 mortality among Black, Latinx, and Asian individuals and overstates the burden among White individuals.

This study is limited by the fact that conclusions comparing inequities in weighted and unweighted populations may change as the epidemic evolves. However, as of July 7, 2020, the CDC’s weighting method remains unchanged.

In summary, the CDC’s presentation of data on race/ethnicity and COVID-19 deaths is misleading, with consequences for resource allocation for mitigating health inequities.^6^ We urge the CDC to drop the misleading weighted counts and publish mortality rates per race/ethnicity group stratified by age, gender, education, and ZIP code characteristics^1^ to adequately equip epidemiologists and policy makers with the data to mitigate inequities.
