# Error in Figure

**DOI:** 10.1001/jamanetworkopen.2020.19768

**Published:** 2020-08-24

In the Research Letter titled “Comparison of Weighted and Unweighted Population Data to Assess Inequities in Coronavirus Disease 2019 Deaths by Race/Ethnicity Reported by the US Centers for Disease Control and Prevention,”^1^ published July 28, 2020, there was an error in panel A of the Figure; 80% (2/5) should have been 80% (4/5). This article has been corrected.^1^
